# Ethnic differences in the mother-son relationship of incarcerated and non-incarcerated male adolescents in the Netherlands

**DOI:** 10.1186/1753-2000-5-23

**Published:** 2011-06-29

**Authors:** Violaine C Veen, Gonneke WJM Stevens, Theo AH Doreleijers, Maja Deković, Trees Pels, Wilma AM Vollebergh

**Affiliations:** 1 Leiden University, Clinical Child and Adolescent Studies, P.O. Box 9555, 2300 RB, Leiden, The Netherlands; 2 Department of Interdisciplinary Social Sciences, Utrecht University, The Netherlands; P.O. Box 80140, 3508 TC, Utrecht, The Netherlands; 3 VU University Medical Centre, Amsterdam, The Netherlands; PO Box 7057, 1007 MB, Amsterdam, The Netherlands; Leiden University, Faculty of Law, Leiden, The Netherlands; 4 Department of Child and Adolescent Studies, Utrecht University, The Netherlands; P.O. Box 80140, 3508 TC, Utrecht, The Netherlands; 5 Verwey-Jonker Institute, Utrecht, The Netherlands; Kromme Nieuwegracht 6, 3512 HG, Utrecht, The Netherlands

**Keywords:** Mother-Child Relations, Ethnic Groups, Prisons

## Abstract

**Background:**

In the Netherlands, youths of Moroccan origin account for a disproportionately large percentage of the population in juvenile justice institutions. Previous research showed that Moroccan adolescents in pre-trial arrest are characterized by less serious offending behavior (i.e., primarily property-based) and lower levels of mental health problems than native Dutch adolescents in pre-trial arrest. To date, little is known about the parent-child relationship of these adolescents. This study examines the mother-son relationships of Moroccan and native Dutch delinquent adolescents and their association with adolescent delinquency.

**Methods:**

In the present study, differences in the mother-son relationship characteristics between families of incarcerated *(N = 129) *and non-incarcerated *(N = 324) *adolescents were examined, and it was analyzed if these differences between incarcerated and non-incarcerated adolescents were the same for Moroccans and native Dutch. Data collection for the incarcerated sample took place from 2006 to 2008. Comparison data were used of interviews conducted with mothers originating from former larger studies in the general Dutch population. Latent Class Analysis was performed in order to identify types of mother-son relationship. Logistic regression analyses were used to identify the relationships between mother-son relationship types, incarceration and ethnicity.

**Results:**

A three class model of mother-son relationship types was found: a low-conflict mother-son relationship type, a high-conflict mother-son relationship type, and a neglectful mother-son relationship type. Compared to the native Dutch adolescents, Moroccans (both in the incarcerated and non-incarcerated population) more often showed a neglectful mother-son relationship type. For Moroccans, no differences in mother-son relationship types were found between the incarcerated and non-incarcerated adolescents, whereas considerable differences occurred between the native Dutch incarcerated and non-incarcerated adolescents.

**Conclusions:**

Our findings indicate that mother-son relationship types of incarcerated Moroccan adolescents and non-incarcerated Moroccan adolescents are rather comparable. These findings are in line with previous studies which revealed the less problematic profile of Moroccan adolescents in pre-trial arrest in the Netherlands compared to native Dutch adolescents in pre-trial arrest.

## Background

In Western societies, youths belonging to an ethnic minority group are disproportionally likely to become engaged in criminal behavior [1-4]. For instance in the US, African-American youth aged 10 to 17 years represent only 15% of their age group, but they account for 26% of juvenile arrest rates and 32% of delinquency referrals to juvenile court. Likewise, in European countries, ethnic minority youths such as West Indians in England and Algerians in France, are clearly overrepresented in juvenile justice institutions [4]. In the Netherlands, youth of Moroccan origin is by far the most conspicuous ethnic group in this respect, with 3% percent in the total population of youth aged 10 to 20 years, but over 25% percent of the total population of incarcerated youth [5].

In a previous study, it was found that Moroccan adolescents in pre-trial arrest in the Netherlands represent a specific offender type [6]. Analyses of criminal record data showed that Moroccan adolescents compared to Dutch native adolescents in pre-trial arrest, were more often incarcerated for property-based offences with or without violence, and less often for violent and sexual offences and for arson. In addition, it is worth noting that in general, the violence used in property offences involving violence, often is relatively light [7]. In about 50% of the cases, the violence used consists of threats in order to obtain property, and in many other cases, light violence is used in order to escape from the crime scene or to flee from the police. Thus, the study examining Moroccan adolescents in pre-trial arrest clearly indicated that a considerable amount of Moroccan adolescents were incarcerated for less serious offences than their Dutch native peers in pre-trial arrest. Moreover, these Moroccan incarcerated adolescents showed lower levels of mental health problems than native Dutch incarcerated adolescents [8]. Compared to Moroccan adolescents in the general population, incarcerated Moroccan adolescents showed higher levels of internalizing and externalizing behavior, but this difference between the general and incarcerated population was much larger for native Dutch adolescents, with native Dutch incarcerated revealing the highest levels of problem behavior. In the current study, we compare the mother-son relationship of Moroccan and native Dutch incarcerated and non-incarcerated male adolescents.

### Parenting and delinquency

There is substantial evidence that a positive parent-adolescent relationship consisting of a combination of parental supervision and support protects adolescents against delinquency [9,10]). In criminological theories, absence of these parenting behaviors is even considered as a strong predictor of juvenile delinquency. Up till now, most research exploring the link between family functioning and juvenile delinquency in general, focuses on the impact of discipline methods and parental monitoring. These studies showed that adolescents who often have conflicts with their parents, or who receive little parental support, are at increased risk of juvenile delinquency [10,11]. In addition, low levels of parental monitoring and high levels of harsh parental disciplining are related to high levels of delinquent behavior in adolescents [12-14].

However, since parental discipline may be perceived differently in groups originating from various cultures, it has also been suggested that the association between parental discipline and delinquency may vary across cultural groups [15]. There is some empirical evidence that the relationship between parental discipline and externalizing behavior is absent or even negative for African-American adolescents, whereas a positive relationship was found for Caucasians [16]. In addition, parental monitoring may have a stronger effect on adolescent delinquency in groups belonging to the ethnic minority than to the ethnic majority. That is, ethnic minority members often live in relatively economically deprived and disorganized neighborhoods, and the protective effect of parental monitoring to adolescent delinquency may be even stronger in such circumstances [14,17]. In accordance, it was found that a lack of parental monitoring was a stronger predictor of adolescent offending for adolescents belonging to ethnic minority groups than for Caucasians [18-20]. However, other studies showed that these ethnic differences are rather small [21,22]. Thus, as findings on ethnic differences in the relationship between parenting and delinquency are inconsistent, general conclusions based on previous findings may be questionable, and further research on this subject is needed.

### Parenting in Moroccan families in the Netherlands

Moroccans belong to one of the largest immigrant groups in the Netherlands. Migration began in the 1960s when Moroccan men were recruited for working in the Dutch labor market. Since then, many of these labor migrants brought their families to the Netherlands and stayed permanently. Nowadays, about 40% of the Moroccan immigrants are born in the Netherlands. Moroccans belong to the least privileged migrant groups in the Netherlands, and public opinion clearly reflects this marginal status [23-25]. The Moroccan culture is characterized by an emphasis on the collective interest of the family, and compliance to parents and older family members [26]. Findings from small-scale, qualitative studies indicate that the parenting style of Moroccan parents on average is characterized by more authoritarian discipline than the parenting style of Dutch parents [27]. Furthermore, it was found that Moroccan parents less often monitor, control and support their children when they reach adolescence [27-29]. Possibly, Moroccan parents consider the upbringing of their children completed at an earlier stage than Dutch parents. A previous large-scale study in Moroccan families in the Netherlands revealed positive correlations between parental affection and discipline, which may imply that parental strictness entails elements of parental interest and warmth in this ethnic group [26]. This may have implications for the relationship between parental discipline and problem behavior. Indeed, this study revealed no association between parental discipline and internalizing problems. The former indicated that there may be considerable differences in the upbringing and the relationship between parenting and delinquency for Moroccan compared to native Dutch families in the Netherlands.

### Limitations of former research

Economic disadvantage is related to negative parenting behaviors such as high levels of strict discipline and low levels of parental warmth [e.g., [17]]. As socio-economic conditions of ethnic minority groups are relatively unfavorable, it is of importance to take this factor into account when examining ethnic differences the association between parenting and juvenile delinquency. Former studies in various ethnic populations have often overlooked socio-economic status as a factor in their analyses. Furthermore, previous research focused on different independent parenting variables (e.g., discipline methods, parental monitoring) in relation to juvenile delinquency, using a variable-centered approach. This is a limitation, as this approach does not capture overall family patterns [30]. Also, most former studies were conducted in the general population using self-report delinquency measures. Since underreporting of (serious) delinquent behavior may be relatively common in self-report studies, and general population studies may not have been able to include large numbers of adolescents who show serious delinquent behavior, previous studies show important limitations in this respect as well [31].

### The present study

This study examines the mother-son relationships of Moroccan and native Dutch delinquent adolescents using an incarcerated sample and comparison samples from the general population. Socio-economic status of the participants is taken into account in all analyses. In the present study, a typological approach is used in which different family types are identified and their association with adolescent delinquency is examined.

Three research questions are addressed in this study. First, what patterns of mother-son relationship characteristics can be identified in a population of incarcerated and non-incarcerated adolescents in the Netherlands? Second, how do patterns of mother-son relationship characteristics in families of incarcerated adolescents compare to patterns of parenting in families of non-incarcerated adolescents? Third, are these differences the same across ethnic groups (Moroccan and native Dutch adolescents)? In line with previous research on the parent-adolescent relationship and juvenile delinquency, it is expected that patterns of mother-son relationship characteristics in families of incarcerated adolescents will more often be characterized by low levels of maternal monitoring and maternal affection and high levels of maternal discipline than the patterns of mother-son relationship characteristics in families of non-incarcerated adolescents. We expect the differences between incarcerated and non-incarcerated boys to be smaller for the Moroccan than for the Dutch adolescents, since our previous studies indicated that Moroccan incarcerated boys in pre-arrest show a less problematic profile than their native Dutch peers. In addition, overall we expect that patterns of mother-son relationship characteristics in Moroccan families are more often characterized by low levels of affection, maternal monitoring and high levels of discipline, compared to the patterns of mother-son relationship characteristics in native Dutch families.

## Methods

### Participants

#### Mothers of incarcerated boys

In the present study, interview data on mothers of incarcerated boys are included, as youth detained under criminal law in juvenile justice institutions merely consist of male detainees, and participating parents in this study were predominantly mothers. The boys were consecutively taken into pre-trial detention in 10 (out of 11) juvenile justice institutions in the Netherlands between May 2006 and February 2008; data collection including interviews with the mothers of these adolescents took place during the same time period. In the Netherlands, criminal legislation for youths applies to persons aged 12 to 18. Pre-trial detention is enforced when a youngster is suspect of an offence, awaiting trial, and if detention is thought to be necessary for the protection of others or the adolescent itself. These adolescents were all suspects of one or more offences.

Eligible for inclusion in the present study were those mothers of whom the son remained in a juvenile justice institution and participated in the study. Dutch mothers had to be able to speak and read Dutch, Moroccan mothers had to be able to speak Moroccan-Arabic. All mothers received a letter containing the aims of the present study. Dutch mothers were contacted by telephone to make an appointment for the interview which took place at the participants' home. Moroccan mothers were sent an introductory letter in Dutch and Moroccan-Arabic and within a couple of weeks a trained Moroccan interviewer visited the parents' home to request them to participate.

Data collection took place at the participants' homes, where questionnaires were handed over to the parent. The questionnaires were filled out by the Dutch mothers. For Moroccan mothers, the questionnaires had been translated into Moroccan-Arabic and to check the accuracy of the translation we performed an independent back translation into Dutch. Since Moroccan parents did not have to be able to read Moroccan-Arabic or Dutch in order to participate in an interview, the questions were read aloud and were filled out by the interviewers. Participants were assured of the confidentiality of their spoken and written responses and data were archived anonymously. Moreover, written informed consent was obtained from the participants. Participants received compensation (a gift certificate). The research protocol was approved by the Ethical Board of the Department of Social Sciences of Leiden University and the Ministry of Justice in the Netherlands. For a more detailed description of the data collection procedure see [8].

Initially, two-hundred seventy-three parents, fathers as well as mothers, were asked to participate in the study. Eighty-four parents refused to participate and 22 parents were not found at home by the interviewers. Thus in total, 167 parents were interviewed, which is a total response rate of 61% (response rate Dutch parents 50%, and response rate Moroccan parents 70%). Of these 167 parental interviews, 129 interviews were conducted with the mother and only these were used in the present study. Sixty-six mothers were of Moroccan origin (i.e., she or the father of her son was born in Morocco) and 63 mothers were of native Dutch origin. Since incomplete participation of the parents may have caused some bias in the findings of the present study, we tested if adolescents, whose parents completed the interview, scored significantly different on self-reported internalizing and externalizing problems as measured by the Youth Self-Report (version 1991), than adolescents whose parents did not participate in the study. No differences were found on internalizing problems (F = 0.392, df = 1, p = 0.53) or externalizing problems (F = 1.106, df = 1, p = 0.29).

#### Moroccan immigrant parents in the general population

Data were used of interviews conducted with mothers originating from a larger study, in which a sample of 1,127 children aged 4 through 18 with at least one parent born in Morocco, were randomly selected from municipal registers of Rotterdam and The Hague. Parents and adolescents were sent an introductory letter in Dutch and Arabic describing the aims of the study and within a couple of weeks a trained Moroccan interviewer visited the respondents' homes to request them to participate. Data collection took place at the participants' homes, where questionnaires were handed over to the parent. The questions were read aloud and filled out by the interviewers. Data collection took place from April 2001 to July 2002. A total of 819 parents participated in the total study (response rate 73%). In the present study mother-reported data of male adolescent participants, aged 13 through 18 (N = 116), were used. The data collection procedure and in- and exclusion criteria are described in detail elsewhere [32].

#### Dutch parents in the general population

Data were used of interviews with mothers that were collected as part of a national research on children/adolescents and their parents, 'Child-rearing in the Netherlands in the 90s'. The families were selected from a larger sample of 10,000 families representative of Dutch population and were first contacted by phone. In the phone conversation the general purpose of the study was explained and it was checked if the parents had an adolescent child. From all contacted families with adolescent children, 53% agreed to participate. Data collection took place from 1993 to 1995 at the participants' homes, where questionnaires were administered individually to adolescents, mothers, and fathers. The sample consisted of 508 families with adolescents aged 12 through 18. In the present study we used mother-reported data of male adolescent participants who were in the age of 13 through 18 (N = 208). The data collection procedure and in- and exclusion criteria are described in further detail elsewhere, see [11].

### Measures

#### Mother-son relationship

To assess maternal parenting practices, two subscales of the Nijmegen Rearing Questionnaire [33] were used: Affection Expression and Discipline. Mothers were asked to indicate on a 6-point scale (1 = highly disagree to 6 = highly agree) whether they agreed with the items. Affection Expression consists of nine items which measure the extent to which the mother shows positive affection towards the child (e.g., 'I often tell my child that I love him/her'). Discipline consists of five items concerning different types of punishment used by the mother (e.g. 'Most of the time, when my child does something he/she is not allowed, I slap him/her', 'I punish my child by sending him/her to his/her room'). Maternal monitoring was measured by means of a six-item instrument on a 4-point scale (1 = nothing to 4 = everything). Mothers were asked to indicate how much they know about, for example, their child's friends, how their child spends free time or how their child spends money [34]. To assess the amount of conflicts between adolescents and their mothers, the Parent-Adolescent Conflict List [35] was used. Mothers were asked to indicate on a 5-point scale (1 = never to 5 = very often) how often they quarrel with their son/daughter about 15 issues (e.g. 'academic achievement', 'curfew', 'home chores', 'son's/daughter's friends', etc.). Reliabilities of the Moroccan-Arabic translations of the scales were comparable to the reliabilities of the Dutch versions. The alphas of the Affection Expression scale were .84 for the Dutch version and .87 for the Moroccan-Arabic version. The alphas of the Discipline scale were .79 for the Dutch version and .86 for the Moroccan-Arabic version. The alphas of the Maternal monitoring scale were .83 for the Dutch version and .91 for the Moroccan-Arabic version.

In order to determine underlying dimensions of the Parent-Adolescent Conflict List, factor analysis was used. Exploratory factor analysis (i.e., Principal Component Analysis) revealed three factors (eigenvalues >1.0) and Varimax rotation (with Kaiser Normalization) showed three distinct factors. Factor 1 represented mother-child conflicts about issues outside the home, whereas Factor 2 represented mother-child conflicts about in-home issues, and Factor 3 represented one item (conflicts about son's girlfriend). The first two factors indicated a 46% explanation of the variance across all 15 items. As Factor 3 consisted of one item only, this item was not further used in the analyses. One item (conflicts about father's/mother's new partner) had low factor loadings on all extracted factors, this item was not retained. Thus, two underlying dimensions of the Parent-Adolescent Conflict List were used as two subscales: Conflicts about issues outside the home and Conflicts about in-home issues. Reliabilities of the two subscales were comparable for Dutch and Moroccan-Arabic versions; the alphas of the Conflicts about issues outside the home subscale were .82 for the Dutch version and .87 for the Moroccan-Arabic version, the alphas of the Conflicts about in-home issues subscale were .70 for the Dutch version and .80 for the Moroccan-Arabic version.

#### Educational level

Parental educational level was scored on a 4-point scale: 0 = elementary school or uncompleted elementary school, 1 = lower level of secondary or vocational education, 2 = medium level of secondary or vocational education and 3 = higher level of vocational education or university. The highest educational level of the father or the mother was used to score educational level of the family. For statistical analyses, the scores were classified into 'low educational level' (0-1), 'moderate educational level' (2) and 'high educational level' (3).

### Statistical analyses

In order to identify different mother-son relationship types within the total sample of native Dutch and Moroccan parents of incarcerated and non-incarcerated adolescents, Latent Class Analysis (LCA) was used. LCA is a statistical method which is used to identify a set of mutually exclusive latent classes that account for the distribution of cases that occur within a cross tabulation of observed variables [36]. In other words, the purpose of a LCA is to find the smallest number of classes of individuals with similar patterns of, in this case mother-son relationships, which can explain associations of a set of variables. The parameters in a LCA model are class specific symptom profiles (which give the probabilities of a set of items for a particular class) and latent class probabilities (which estimate the likelihood for individuals to belong to each of the classes). Individuals are classified to the group with their highest class probability. The number of latent classes is determined by testing the goodness of fit of models with N latent classes using the Vuong-Lo-Mendell-Rubin likelihood ratio test and goodness of indices such as the Akaike Information Criterion, the Bayesian Information Criterion and Entropy. Latent Class Analyses were conducted in the software package Mplus version 5. To test the representation of native Dutch and Moroccan mothers of incarcerated and non-incarcerated adolescents in each of the classes, Chi-square tests were used. In order to identify the relationship of incarceration of the child and ethnicity on each of the mother-son relationship types, logistic regression analyses were conducted. Interaction effects between incarceration and ethnicity on mother-son relationships were tested using logistic regression analyses.

## Results

### Descriptives

Table 1 shows descriptive statistics and differences in the mean scores on the mother-son relationship characteristics between each subsample. These characteristics differed significantly between the samples: Discipline (F = 20.847, df = 3, *p *< 0.01); Affection Expression (F = 4.951, df = 3, *p *< 0.01); Monitoring (F = 8.529, df = 3, *p *< 0.01); Conflicts about issues outside the home (F = 6.971, df = 3, *p *< 0.01); Conflicts about in-home issues (F = 24.561, df = 3, *p *< 0.01).

**Table 1:** Descriptives and mean scores (SD) on the mother-son relationship characteristics for each subsample

	ND N = 208	NM N = 116	ID N = 63	IM N = 66	Range (Min - Max)	Skew-ness	Kurtosis
Affection Expression	38.75^a ^(7.57)	36.38^b ^(10.30)	35.90^b,c ^(8.84)	40.47^a ^(8.27)	45 (9 - 54)	-.570	.127
Discipline	14.67^a ^(5.16)	18.36^b ^(7.65)	17.14^b^ (6.30)	21.41^c^ (7.97)	25 (5 - 30)	-.044	-.909
Monitoring	19.82^a ^(2.22)	18.20^b,c ^(4.28)	17.58^c ^(3.90)	19.07^a,b ^(5.42)	18 (6 - 24)	-.967	1.242
Conflicts issues outside home	14.56^a ^(4.66)	15.13^a ^(6.44)	17.49^b ^(6.26)	17.56^b ^(7.77)	28 (7 - 35)	.701	.169
Conflicts in-home issues	13.72^a ^(3.82)	11.59^b ^(4.62)	12.89^a,c ^(4.11)	8.94^d ^(4.01)	24 (6 - 30)	.575	.105

Note. ND = Non-incarcerated Dutch, NM = Non-incarcerated Moroccan, ID = Incarcerated Dutch, IM = Incarcerated Moroccan; a,b,c,d Different superscripts refer to significant differences (p < 0.05) between the groups (within rows), tested by means of MANOVA.

### Mother-son relationship types

**Figure 1:**
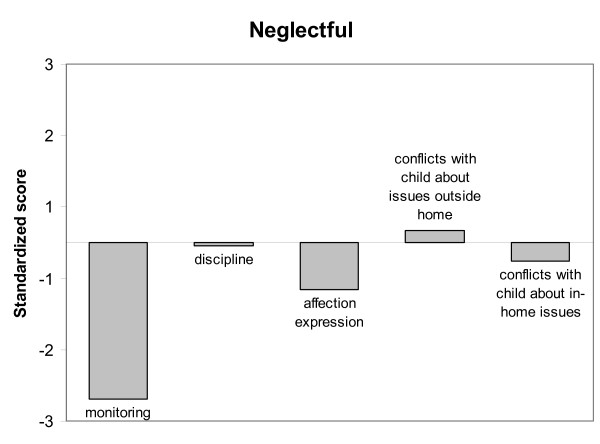
**Neglectful mother-son relationship type**.

**Figure 2:**
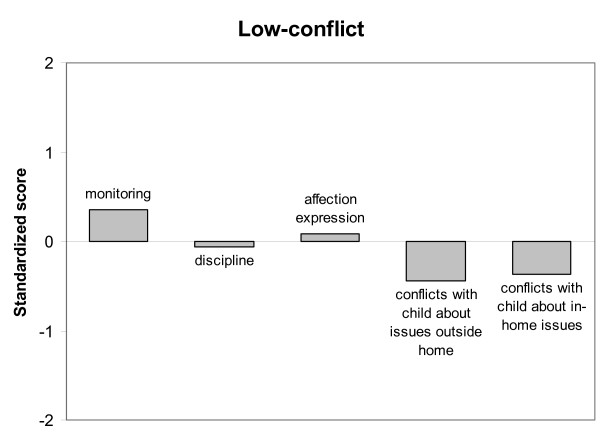
**Low-conflict mother-son relationship type**.

**Figure 3:**
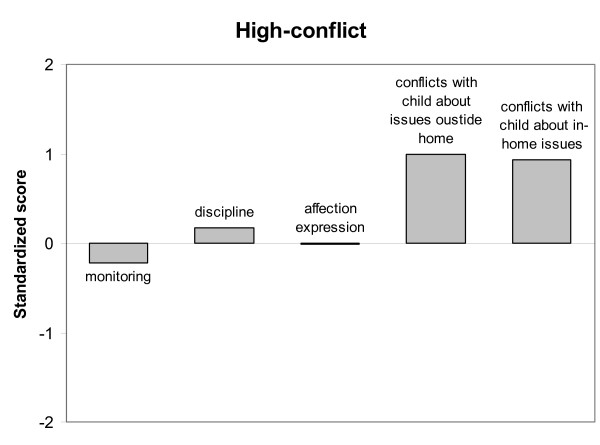
**High-conflict mother-son relationship type**.

Latent Class Analysis was performed based on mother-son relationship characteristics using the standardized scores (Z-scores) of each participant on the following scales: Monitoring, Discipline, Affection Expression, Conflicts about issues outside home and Conflicts about in-home issues. LCA showed a significant three-class model for the total group of native Dutch and Moroccan mothers (of incarcerated adolescents and non-incarcerated adolescents), which was the best fitting LCA-solution according to the Vuong-Lo-Mendell-Rubin likelihood ratio test (*p *< 0.05). The Akaike Information Criterion and the Bayesian Information Criterion were both lower for the three-class model (AIC = 6241.991 and BIC = 6332.541) than for the two-class model (AIC = 6308.574 and BIC = 6374.428), indicating a more parsimonious solution. A four-class model did not improve the LCA-solution. Also, the Entropy of the three-class model was satisfactory (0.75), indicating a good model as well. The average class probabilities were high (.84 - .90), which indicated that the participants were properly classified to their latent class. Figure 1 shows the standardized scores on each mother-son relationship characteristic for Class 1. Class 1 (8% of 453 participants) was characterized by extreme low scores on monitoring, indicating a very low awareness in mothers of their child's affairs, low scores on affection expression and average scores on conflicts with child about issues outside the home. This class was also characterized by slightly below average scores on conflicts with the child about in-home issues and almost average scores on discipline. Class 1 therefore, could be termed *neglectful mother-son relationship*. Class 2 (64% of 453 participants) was characterized by above average scores on monitoring, below average scores on conflicts with the child (on in-home issues and issues outside the home) and almost average scores on affection expression and discipline. Class 2 therefore was termed *low-conflict mother-son relationship*, see Figure 2. Class 3 (28% of 453 participants) was characterized by high scores on conflicts with the child about in-home issues and issues outside the home, somewhat above average scores on discipline, but slightly below average scores on monitoring and average scores on affection expression. Class 3 was termed *high-conflict mother-son relationship*, see Figure 3.

### Mother-son relationship types and population samples

The neglectful mother-son relationship type was predominantly found in the Moroccan incarcerated population (15,2%), the Moroccan non-incarcerated population (12,9%), and in the native Dutch incarcerated population (9,5%), see Table 2. In the native Dutch non-incarcerated population, only 1,0% of the mothers reported this mother-son relationship type (Chi = 24.3, df = 3, *p *= 0.00). The low-conflict mother-son relationship type was found in 75,5% of the Dutch families in the non-incarcerated population and in 57,1% of the Dutch families with an incarcerated son (Chi = 11.2, df = 3, *p *= 0.01). The high-conflict mother-son relationship type was predominantly (33,3%) found in the Dutch incarcerated population (Chi = 3.1, df = 3, *p *= 0.38).

**Table 2:** Representation of mother-son relationship types in population samples

	Neglectful		Low-conflict		High-conflict	
	Dutch	Moroccan	Dutch	Moroccan	Dutch	Moroccan
Non-incarcerated	1.0%	12.9%	75.5%	62.1%	23.6%	25.0%
Incarcerated	9.5%	15.2%	57.1%	63.6%	33.3%	21.2%
Total	3.0%	13.7%	71.2%	62.6%	25.8%	23.6%

Logistic regression analyses were conducted to identify the direct relationships between ethnicity and incarceration of the child with the three mother-son relationship types, see Table 3. Initially, our bivariate analyses showed that incarcerated boys were more likely to have a neglectful mother-son relationship and that non-incarcerated boys were more likely to have a low-conflict relationship with their mother. When ethnicity, educational level of the parents, single-parenting and age of the child were included in the multiple regression models, these effects did not remain significant. Next, ethnicity was significantly related to the neglectful mother-son relationship type. Mothers of Moroccan origin were (nearly five times) more likely to report this type of mother-son relationship, and this effect (OR = 3.91, CI 1.45-10.56, *p *= 0.01) remained significant when all other variables were included in the multiple regression model.

**Table 3:** Main- and interaction effects of incarceration and ethnicity to mother-son relationship types

	Neglectful				Low-conflict				High-conflict			
	**Bivariate**		**Multiple**^**1**^		**Bivariate**		**Multiple**^**1**^		**Bivariate**		**Multiple**^**1**^	
**Factor**	**OR**	**CI 95%**	**OR**	**CI 95%**	**OR**	**CI 95%**	**OR**	**CI 95%**	**OR**	**CI 95%**	**OR**	**CI 95%**

Incarceration	2.56*	1.25-5.23	1.71	0.79-3.71	0.63*	0.41-0.97	0.70	0.44-1.10	1.17	0.74-1.87	1.27	0.78-2.08
Ethnicity	5.24*	2.31-11.89	3.91*	1.45-10.56	0.68	0.46-1.01	0.87	0.54-1.41	0.89	0.57-1.38	0.73	0.43-1.23
Ethnicity × incarceration	-	-	0.14*	0.02-0.92	-	-	2.22^NB^	0.93-5.33	-	-	0.52	0.20-1.35

Note. *p < 0.05; ^NB^p = 0.07; ^1^Multiple logistic regression analyses are controlled for age of the child, educational level of the parents, single-parenting; Ethnicity (native Dutch = 0, Moroccan = 1, reference category = native Dutch), Incarceration (non-incarcerated = 0, incarcerated = 1, reference category = non-incarcerated).

In addition, interaction effects of incarceration and ethnicity to the three mother-son relationship types were tested, see Table 2. Only for Dutch native families, incarcerated boys more often had a neglectful mother-son relationship than boys who were not incarcerated (OR = 7.27, CI = 1.24-42.56, *p *= 0.03), whereas no such differences were found for families of Moroccan origin (OR = 1.13, CI = 0.46-2.76, *p *= 0.79). Similarly, incarceration of the child was significantly related to a low-conflict mother-son relationship in Dutch families (OR = 0.47, CI = 0.25-0.89, *p *= 0.02), i.e., mothers of non-incarcerated children more often reported a low-conflict mother-son relationship type. This effect was not found in families of Moroccan origin (OR = 1.06, CI = 0.55-2.05, *p *= 0.86).

## Discussion

The purpose of this study was to examine differences in patterns of mother-son relationship characteristics between families of incarcerated and non-incarcerated adolescents, and to examine these differences across ethnic groups (Moroccan and native Dutch families). Using Latent Class Analysis, a three class model of mother-son relationships was found: a *low-conflict *mother-son relationship type, a *high-conflict *mother-son relationship type, and a *neglectful *mother-son relationship type. It was found that the low-conflict mother-son relationship type, characterized by relatively low levels of mother-son conflicts, above average monitoring, and average affection and discipline, was most common in all populations (i.e., incarcerated, non-incarcerated, native Dutch and Moroccan families), but was found most often in native Dutch families of non-incarcerated boys. The high-conflict mother-son relationship type, characterized by high levels of mother-son conflicts and average affection was found in about a quarter of all populations, and was slightly more prevalent in the Dutch incarcerated sample than in the other samples. Finally, the neglectful mother-son relationship, which indicated a low awareness in mothers of their child's affairs and little affection, was found in a small percentage of all populations, but was virtually absent in the Dutch native non-incarcerated sample.

In line with previous research on the parent-adolescent relationship and juvenile delinquency, it was expected that mother-son relationship types in families of incarcerated adolescents would be more often characterized by low levels of maternal monitoring and maternal affection and high levels of maternal discipline than these types in families of non-incarcerated adolescents. Indeed, the neglectful mother-son relationship was less likely to be reported by native Dutch mothers of non-incarcerated adolescents than by native Dutch mothers of incarcerated adolescents. However, in contrast to our expectations, a mother-son relationship type characterized by high levels of maternal *discipline *was not found in the present study. For Moroccan families, the absence of a mother-son relationship type comprising high disciplining, may reflect a change in maternal parenting behavior over time, i.e., when their children reach adolescent age. For instance, previous small-scale research showed that Moroccan parents less often monitor and control their children when they reach adolescence [27-29]. Finally, the high-conflict mother-son relationship type, which indicated the presence of mother-son conflicts, was only slightly more prevalent among families of incarcerated adolescents.

For Moroccan families no associations were found between the neglectful mother-son relationship type and incarceration, but a Moroccan background as such was found to be associated with the neglectful mother-son relationship type, even when taking the educational level of the parents, single-parenting, and incarceration of the child into account. In fact, the neglectful mother-son relationship type was clearly present in a small percentage (14%) of Moroccan families. These findings are in line with previous small-scale, qualitative research, which showed that the mother-son relationship of Moroccan mothers and their sons is sometimes characterized by little support and control [27,29]. This may be explained by the fact that Moroccan parents consider the upbringing of their sons completed at an earlier age than Dutch parents.

This study is the first to examine ethnic differences in the association between mother-adolescent relationships and juvenile delinquency using an incarcerated sample and comparison samples of non-incarcerated adolescents. Some limitations of this study should be noted. First, the cross-sectional nature of the study makes it difficult to examine causal pathways regarding ethnic differences in mother-son relationships and juvenile delinquency. Second, the identification of mother-son relationship types was based on self-report instruments and may to some extent have been subject to social desirability (i.e., biased self-presentation). However, in contrast to most studies on ethnic differences in mother-son relationships and juvenile delinquency, a four-group design was used. As such, it was possible to control for a general social desirability tendency by making comparisons between Moroccan families of incarcerated adolescents and Moroccan families of non-incarcerated adolescents. Third, in the present study only mothers were included. This means that the role of the father in the parent-child relationship was not examined. Since maternal and paternal parenting behaviors may have differential effects on adolescent behavior, future research should examine these effects on delinquent behavior across ethnic groups. In addition, it would also be desirable to include data on the parent-child relationship from multiple sources, such as adolescent-reports and father-reports. Finally, in the present study, data from different comparison samples were used in order to examine associations between mother-son relationship types, ethnicity and incarceration. It should be noted that the data were collected at different points in time, with data on non-incarcerated boys preceding the data on the incarcerated boys with five to six years (i.e., non-incarcerated Moroccan sample) and with 11 to 13 years (i.e., non-incarcerated native Dutch sample). This means that the time differences between data collection in the different samples may have affected findings.

## Conclusions

Our findings indicated that mother-son relationship types of incarcerated Moroccan adolescents and non-incarcerated Moroccan adolescents are rather comparable. This is in line with previous studies which revealed that Moroccan adolescents in pre-trial arrest in the Netherlands represent an offender type characterized by less serious offending behavior and less mental health problems than native Dutch adolescent offenders. Finally, in the present study, a neglectful mother-son relationship was found to be more prevalent among Moroccan than among native Dutch families: in one-seventh of the Moroccan families a neglectful mother-son relationship was reported. It seems likely that boys brought up in these families are at an increased risk of a problematic development, as could be reflected in the substantial overrepresentation of this group in youth detention. This implies that support is warranted for these boys.

## Authors' contributions

VV participated in the design of the study, carried out the data-collection, performed the statistical analysis and drafted the manuscript. GS conceived of the study, and participated in its design and coordination, provided comparison data and helped to draft the manuscript. TD helped to draft the manuscript. MD provided comparison data and critically revised the manuscript for important intellectual content. TP critically revised the manuscript for important intellectual content. WV helped conceive of the study, participated in its design and coordination and helped to draft the manuscript. All authors read and approved the final manuscript.

## Authors' information

Violaine Veen is an Assistant Professor at the Department of Clinical Child and Adolescent Studies of Leiden University. Her research interests include the development of delinquent behavior and risk factors of delinquency. Her Ph.D. research was to examine the mechanisms leading to delinquent behavior in Moroccan youth, one of the largest ethnic minority groups in the Netherlands.

Gonneke Stevens is an Assistant Professor at the Department of Interdisciplinary Social Sciences of Utrecht University. She received her Ph.D. in Child & Adolescent Psychiatry at the Erasmus MC/Sophia, Rotterdam. Her research interests concern the psychological development of immigrant children and adolescents.

Theo Doreleijers is professor of child and adolescent psychiatry at the VU University Medical Centre and training professor at the Academic Centre of Child and Adolescent Psychiatry de Bascule in Amsterdam, and he is professor of forensic psychiatry at the Faculty of Law, Leiden University. He is also chairman of EFCAP, the European Association for Forensic Child and Adolescent Psychiatry and Psychology.

Maja Deković is full professor at the Department of Child and Adolescent Studies of the Utrecht University. Her research interests include development of problem behavior, parent-child relationships, family interaction and effects of family-based interventions.

Trees Pels is professor at the Department of Psychology and Education of VU University in Amsterdam, and senior researcher at the Verwey-Jonker Institute in Utrecht, where she leads the research programme Diversity. Her field of study is the socialization and development of minority children at home and at school and the interaction between their families, peer group and other socializing agents and institutions.

Wilma Vollebergh is full professor at the Department of Interdisciplinary Social Sciences at the Faculty of Social and Behavioural Sciences of Utrecht University. She is heading the research programme on Youth in Changing Cultural Contexts. Her research interests include mental health, risk behavior and substance (ab)use in adolescents.

## Competing interests

The authors declare that they have no competing interests.

## References

[B1] Engen RL Steen S Bridges GS Racial disparities in the punishment of youth: A theoretical and empirical assessment of the literature Social Problems 2002 49 2 194 220 10.1525/sp.2002.49.2.194

[B2] Rodney HE Tachia HR Over-representation of minorities in the juvenile justice system: Three counties in rural Texas Federal Probation 2004 68 3 44 48

[B3] Sickmund M Sladky TJ Kang W Census of Juveniles in Residential Placement Databook 2008

[B4] Tonry M Tonry M Ethnicity, crime, and immigration Ethnicity, crime, and immigration: comparative and cross-national perspectives 1997 Chicago: The University of Chicago Press

[B5] Boendermaker L Jongeren in justitiële behandelinrichtingen [Youths in juvenile justice treatment institutions] 1995

[B6] Veen VC Stevens GWJM Doreleijers TA Vollebergh WA Moroccan adolescent suspect offenders in the Netherlands: Ethnic differences in offender profiles Psychology, Crime and Law 2011 1 17 iFirst

[B7] Van der Vinne H Geweld in vermogensdelicten: Een dieptestudie op basis van de WODC-strafrechtmonitor [Violence in property offences: An in-depth study based on the WODC Criminal law monitor] 1999

[B8] Veen VC Stevens GWJM Doreleijers TA van der Ende J Vollebergh WA Ethnic differences in mental health among incarcerated youths: Do Moroccan immigrant boys show less psychopathology than native Dutch boys? European Child & Adolescent Psychiatry 2010 19 5 431 440 10.1007/s00787-009-0073-0 20449708 PMC2865629

[B9] Deković M Wissink IB Meijer AM The role of family and peer relations in adolescent antisocial behaviour: comparison of four ethnic groups J Adolesc 2004 27 5 497 514 10.1016/j.adolescence.2004.06.010 15475043

[B10] Gorman-Smith D Tolan PH Henry DB A developmental-ecological model of the relation of family functioning to patterns of delinquency Journal of Quantitative Criminology 2000 16 2 169 198 10.1023/A:1007564505850

[B11] Deković M Parent-Adolescent Conflict: Possible Determinants and Consequences International Journal of Behavioral Development 1999 23 4 977 1000 10.1080/016502599383630

[B12] Lahey BB Van Hulle CA D'Onofrio BM Rodgers JL Waldman ID Is parental knowledge of their adolescent offspring's whereabouts and peer associations spuriously associated with offspring delinquency? Journal of Abnormal Child Psychology 2008 36 6 807 823 10.1007/s10802-008-9214-z 18214666

[B13] Patterson GR Stouthamer-Loeber M The correlation of family management practices and delinquency Child Development 1984 55 1299 1307 10.2307/1129999 6488958

[B14] Sampson RJ Laub JH Urban Poverty and the Family Context of Delinquency - a New Look at Structure and Process in a Classic Study Child Development 1994 65 2 523 540 10.2307/1131400 8013238

[B15] Rudy D Grusec JE Correlates of authoritarian parenting in individualist and collectivist cultures and implications for understanding the transmission of values Journal of Cross-Cultural Psychology 2001 32 2 202 212 10.1177/0022022101032002007

[B16] Deater-Deckard K Dodge KA Bates JE Pettit GS Physical discipline among African American and European American mothers: Links to Children's Externalizing Behaviors Developmental Psychology 1996 32 6 1065 1072

[B17] Barnett MA Economic disadvantage in complex family systems: Expansion of family stress models Clinical Child and Family Psychology Review 2008 11 3 145 161 10.1007/s10567-008-0034-z 18491229 PMC4095799

[B18] Bird HR Canino GJ Davies M Zhang HY Ramirez R Lahey BB Prevalence and correlates of antisocial behaviors among three ethnic groups Journal of Abnormal Child Psychology 2001 29 6 465 478 10.1023/A:1012279707372 11761281

[B19] Cernkovich SA Giordano PC Family relationships and delinquency Criminology 1987 25 2 295 321 10.1111/j.1745-9125.1987.tb00799.x

[B20] Smith C Krohn MD Delinquency and Family-Life among Male-Adolescents - the Role of Ethnicity Journal of Youth and Adolescence 1995 24 1 69 93 10.1007/BF01537561

[B21] Vazsonyi AT Pickering LE The importance of family and school domains in adolescent deviance: African American and Caucasian youth Journal of Youth and Adolescence 2003 32 2 115 128 10.1023/A:1021857801554

[B22] Wissink IB Deković M Meijer AM Parenting behavior, quality of the parent-adolescent relationship, and adolescent functioning in four ethnic groups Journal of Early Adolescence 2006 26 2 133 159 10.1177/0272431605285718

[B23] Gijsberts M SCP, WODC, CBS Opvattingen van allochtonen en autochtonen over de multi-etnische samenleving [Beliefs of immigrants and native Dutch on the multi-ethnic society] Jaarrapport integratie 2005 [Year Report Integration 2005] 2005 Den Haag 189 206

[B24] Hagendoorn L Sniderman P Experimenting with a national sample: a Dutch survey of prejudice Patterns of Prejudice 2001 35 4 19 31 10.1080/003132201128811269 18268831

[B25] Van Praag CS Dagevos J, Gijsberts M, van Praag C Wederzijdse beeldvorming [Mutual conceptualization] Rapportage minderheden 2003: Onderwijs, arbeid en sociaal-culturele integratie [Minorities Report 2003: Education, labour and socio-cultural integration] 2003 Den Haag: SCP 363 392

[B26] Stevens GWJM Vollebergh WAM Pels TVM Crijnen AAM Problem behavior and acculturation in Moroccan immigrant adolescents in the Netherlands - Effects of gender and parent-child conflict Journal of Cross-Cultural Psychology 2007 38 3 310 317 10.1177/0022022107300277

[B27] Pels T Nijsten C Hagendoorn L, Veenman J, Vollebergh WAM Myths and realities of diversity in child rearing and parent-child relations: Non-indigenous compared to indigenous families in the Netherlands Structural integration and cultural orientations in indigenous and non-indigenous Dutch citizens 2003 Aldershot: Ashgate

[B28] Stevens GWJM Vollebergh WAM Pels TVM Crijnen AAM Parenting and internalizing and externalizing problems in Moroccan immigrant youth in the Netherlands Journal of Youth and Adolescence 2007 36 5 685 695 10.1007/s10964-006-9112-z

[B29] Pels T De Haan M Continuity and change in Moroccan socialization: A review of the literature on socialization in Morocco and among Moroccan families in the Netherlands 2003

[B30] Mandara J The typological approach in child and family psychology: A review of theory, methods, and research Clinical Child and Family Psychology Review 2003 6 2 129 146 10.1023/A:1023734627624 12836581

[B31] Piquero AR MacIntosh R Hickman M The validity of a self-reported delinquency scale - Comparisons across gender, age, race, and place of residence Sociological Methods & Research 2002 30 4 492 52921709825

[B32] Stevens GWJM Pels T Bengi-Arslan L Verhulst FC Vollebergh WA Crijnen AA Parent, teacher and self-reported problem behavior in The Netherlands: comparing Moroccan immigrant with Dutch and with Turkish immigrant children and adolescents Social Psychiatry and Psychiatric Epidemiology 2003 38 10 576 85 10.1007/s00127-003-0677-5 14569425

[B33] Gerris JRM Boxtel DAAM Verhulst AA Jansenss JMAM van Zutphen RAH Felling AJA Parenting in Dutch families 1993

[B34] Brown BB Mounts N Lamborn SD Steinberg L Parenting practices and peer group affiliation in adolescence Child Development 1993 64 467 482 10.2307/1131263 8477629

[B35] Noom MJ Dekovic M Hofer M, Youniss J, Noack P Family interaction as a context for the development of adolescent autonomy Verbal interaction and development in families with adolescents 1998 Stamford, CT: Ablex 109 125

[B36] McCutcheon AL Latent Class Analysis 1987 Newbury Park, CA: Sage

